# Effects of the Health Belief Model (HBM)-Based Educational Program on the Nutritional Knowledge and Behaviors of CABG Patients

**Published:** 2016-10-03

**Authors:** Sarallah Shojaei, Roohollah Farhadloo, Afsaneh Aein, Mostafa Vahedian

**Affiliations:** 1 Nekoei Hospital, Qom University of Medical Sciences, Qom, Iran.; 2 Qom University of Medical Sciences, Qom, Iran.; 3 Health Education and Health Promotion, School of Health, Iran University of Medical Sciences, Tehran, Iran.; 4 Clinical Research Development Center, Qom University of Medical Sciences, Qom, Iran.

**Keywords:** *Coronary artery bypass*, *Knowledge*, *Perception*, *Behavior*

## Abstract

**Background:** Reducing blood pressure through diet decreases the possibility of heart attacks, and lowering blood cholesterol can reduce the risk of coronary artery disease. The aim of the present study was to examine the effects of education based on the Health Belief Model on the dietary behavior of patients following coronary artery bypass graft surgery (CABG) at the Heart Surgery Department of Shahid Beheshti Hospital of Qom.

**Methods:** In this semi-experimental clinical trial, data were collected on 64 patients, at an average age of 59.9 ± 7.26 years in the intervention group and 58.5 ± 7.6 years in the control group. Seventy percent of the study subjects were male and 30% were female. Intervention and control groups were given a questionnaire, comprising 56 questions in 5 parts. The educational intervention was aimed at creating perceived susceptibility and perceived severity in the intervention group. After 1 month. Both groups were tested, and the resulting data were analyzed to investigate the effects of the educational intervention on the nutritional knowledge and behavior of the patients.

**Results:** According to the results, educational intervention caused a significant increase in the mean scores of knowledge (p value = 0.001), perceived severity (p value = 0.007), and perceived benefits and barriers (p value = 0.003) in the intervention group but did not cause a significant increase in the mean score of nutritional behavior (p value = 0.390).

**Conclusion:** Education based on the Health Belief Model seems to be effective in improving nutritional knowledge, but more consistent and comprehensive educational programs are necessary in order to change behavior and improve nutritional behavior.

## Introduction

Cardiovascular disease (CVD) is among the main causes of deaths around the world and according to the World Health Organization’s prediction, CVD will cause 25 million deaths in 2020.^1^ In addition, CVD-especially in the elderly-accounts for a fifth of disabilities.^2^ The prevalence of CVD is on the rise in developing countries,^3^ and it is an increasing health threat in Eastern Mediterranean and Middle East countries.^4^ It has been suggested that a lack of knowledge regarding CVD risk factors and some misconceptions can contribute to an unhealthy lifestyle,^5^ which can consequently lead to an increased CVD prevalence (Harrell TK, Horton NN, King DS, Wofford MR. Assessment of cardiovascular risk factor awareness and accentuation of early detection among African-American college students. American Journal of Hypertension 2003;16(S1):244A-5A.).^6^ Health promotion programs aimed at reducing risky behaviors, promoting a healthy lifestyle, and controlling CVD risk factors may reduce the prevalence of CVD. Also, preventing the progression of the disease may enable patients to return to work and enjoy a normal life, which in turn could lessen the burden of the disease.^7^

The Health Belief Model (HBM) was developed initially in the early 1950s by social psychologists. Since then, it has been widely drawn upon to explain alterations in health-related behaviors and to guide health behavior interventions. This model is employed to help change behaviors vis-à-vis CVD, high blood pressure, nutritional problems, contraceptive pills, and breast self-examinations (Shojaeizadeh D. Models of Behavior study in health education. Ministry of Health and Medical Education; Tehran, 1998. p. 15-30.). The main constructs of the HBM comprise: 1) perceived threat, which consists of A) perceived susceptibility: a person’s subjective perception of the risk of acquiring a disease and B) perceived severity: a person’s feelings about the seriousness of contracting a disease; 2) perceived benefits: a person’s perception of the effectiveness of various actions available to reduce the threat of a disease; 3) perceived barriers: a person’s belief about the potential negative aspects of taking a particular health action; and 4) cue to action:  internal or external cues that determine a person’s readiness for action and trigger the decision-making process. According to the HBM, a change in belief precedes a change in behavior.^8^ Following a healthy diet is an effective factor in the preventive measures for CVD, as is the case in cardiac rehabilitation after coronary artery bypass graft surgery (CABG).^9^ This can be attained by preventing unhealthy behaviors through education and training.^7^ Accordingly, the present study aimed to test the effectiveness of a proper nutrition education in an Iranian sample of CABG patients in the prevention of further complications related to this condition.

## Methods

The current study was a randomized controlled clinical trial on patients undergoing CABG at the Heart Surgery Department of Shahid Beheshti, Qom, Iran. We sought to evaluate the patients’ level of knowledge about proper nutrition based on the HBM and assess the effectiveness of a proper nutrition education as regards the constructs of the HBM-namely perceived susceptibility, perceived severity, perceived benefits and barriers, and cues to action. Since the intervention in this study was in the form of education by the researchers, blinding was impossible. After visiting the heart department and analyzing the patients’ records, we found the prospective participants who met the inclusion criteria. Then, the details of the study (i.e., the method and aim of the study; the timing, benefits, and potential dangers of the study; confidentiality; and ethics) were explained to the prospective participants, and they were asked to read and sign consent forms and set an appointment with the researchers for interviews. Thereafter, the final files were numbered and the even numbers were assigned to the intervention group and the odd ones to the control group. The required information was obtained from the intervention and control groups through a 5-part questionnaire (pretest). The nutrition education intervention was conducted based on the perceived susceptibility and perceived severity of CVD and with a focus on the benefits of a healthy diet and the removal of barriers according to nutrition books and cardiologists’ guidance through 2 face-to-face 1-hour training sessions in the hospital (in the presence of the patients’ companions and those who had a role in their diet). Additionally, nutrition pamphlets were given to the patients in order to increase the persistence of information. There was a week’s interval between the 2 sessions, and the control group received no treatment. A post test was conducted on both groups 1 month later. Finally, the data were analyzed using SPSS, version 16, in order to assess the effects of the education intervention on the knowledge and behavior of the participants.

The data collection instrument was a 5-part questionnaire containing 56 questions based on the HBM. The 1st part concerned individual differences, the 2nd part dealt with the patients’ knowledge of CVD and proper nutrition, the 3rd part covered perceived threat, the 4th part inquired about perceived benefits and barriers, and the 5th part probed into the patients’ behavior. The validity of the questionnaire had been proved in previous similar studies,^9^ and its Cronbach’s α reliability coefficient was 0.78 in the knowledge, 0.85 in the perceived severity, 0.57 in the perceived benefits, and 0.72 in the behavior parts.

In the current study, the confidence level and the statistical power used for calculating the sample size were 95% and 80%, respectively. The number of participants in each group was 28, but 32 participants were considered because there could be a 10% dropout rate. The mean scores and standard deviation of the data were calculated, and the independent sample T-test and T-test were used to analyze the data. The results of the Kolmogorov-Smirnov test showed that the distribution of the demographic variables of the participants was normal and, therefore, parametric tests were utilized to analyze the data. A significance level of 0.05 was considered in all the parametric analyses.

## Results

A total of 64 eligible and accessible patient were randomly divided into either control or intervention group equally. According to the results, the mean age of the patients in the intervention group was 59.9 ± 7.26, with a minimum of 42 and a maximum of 72 years, and the mean age of the patients in the control group was 58.5 ± 7.6, with a minimum of 46 and a maximum of 70 years. In addition, 70% of the study subjects were male and 30% were female. A comparison of the 2 groups in terms of their basic demographic characteristics and the data on the CABG patients are depicted in Table 1. The results of the χ^2^ test did not show any significant differences between the 2 study groups regarding the demographic variables. The results are expressed as significant at a p value = 0.055) (Table 1).

The mean scores of the nutritional knowledge level of the CABG patients in the control and intervention groups showed no significant differences before the intervention (p value = 0.652). The mean score and standard deviation of the nutritional knowledge level in the intervention group were 12.20 and 2.47 and those in the control group were 9.80 and 2.10. However, the mean scores of the nutritional knowledge level of the patients in the control and intervention groups showed a significant difference after the intervention (p value = 0.001). This showed that education had been effective in improving the patients’ nutritional knowledge level. In addition, the mean score and standard deviation of the nutritional knowledge level were 9.28 and 2.80 before the intervention and 9.80 and 2.10 after the intervention in the control group, which showed no significant differences before and after the intervention (p value = 0.210). The mean score and standard deviation of the nutritional knowledge level were 9.00 and 2.15 before the intervention and 12.20 and 2.47 after the intervention in the intervention group, which showed a significant difference before and after the intervention (p value = 0.001) (Table 2). 

The mean scores of perceived severity in the CABG patients in the control and intervention groups showed no significant differences before the intervention (p value = 0.541). Nonetheless, the mean score and standard deviation of perceived severity were 21.80 and 4.30 in the intervention group and 17.90 and 4.60 in the control group after the intervention, which showed a significant difference between the 2 groups after the intervention (p value = 0.001). In addition, the mean score of perceived severity in the control group showed no significant difference before and after the intervention (p value = 0.913), while the mean score of perceived severity in the patients in the intervention group showed a significant difference before and after the intervention (p value = 0.007) (Table 2).

The mean score and standard deviation of perceived benefits and barriers in the CABG patients were 14.60 and 3.40 in the intervention group and 14.20 and 3.11 in the control group before the intervention, which showed no significant difference (p value = 0.650). Nevertheless, after the intervention, these figures were 17.80 and 4.70 in the intervention group and 14.50 and 3.20 in the control group, which indicated a significant difference between the 2 groups after the intervention (p value = 0.001). Furthermore, perceived benefits and barriers in the patients in the control group before the intervention indicated no significant differences compared to those after the intervention (p value = 0.752). However, perceived benefits and barriers in the patients in the intervention group indicated significant differences after the intervention (p value = 0.003) (Table 3).

The statistical analyses showed that the mean score and standard deviation of dietary behavior in the CABG patients before the intervention were 27.00 and 3.10 in the intervention group and 26.20 and 2.90 in the control group, which showed no significant differences between the 2 groups (p value = 0.080). However, after the intervention, these figures were 27.30 and 3.22 in the intervention group and 25.20 and 3.00 in the control group, which indicated a significant difference between the 2 groups. The mean score and standard deviation of dietary behavior in the patients in the control group were 26.20 and 2.90 before the intervention and 25.20 and 3.00 one month later, which indicated no significant differences (p value = 0.481). Also, the mean score and standard deviation of dietary behavior in the patients in the intervention group were 27.00 and 3.10 before the intervention and 27.30 and 3.22 after the intervention, which indicated no significant differences (p value = 0.394) (Table 3).

**Table 1:** Patients’ characteristics*

	Intervention Group	Control Group	P value
Age (y)	59.9±7.26	58.5±7.6	0.451
Gender			0.055
Male	26 (81.2)	19 (59.4)	
Female	6 (18.8)	13 (40.6)	
Smoking			0.614
Yes	11 (34.4)	13 (40.6)	
No	21 (65.6)	19 (59.4)	
Family history of heart disease			0.612
Yes	14 (43.8)	12 (37.5)	
No	18 (56.2)	20 (62.5)	
Diabetes mellitus			0.303
Yes	10 (31.2)	14 (43.8)	
No	22 (68.8)	18 (56.2)	
Hypertension			0.450
Yes	15 (46.9)	12 (37.5)	
No	17 (53.1)	20 (62.5)	
Hyperlipidemia			0.110
Yes	7 (21.9)	13 (40.6)	
No	25 (78.1)	19 (59.4)	

* Data are presented as means±SD or numbers (%)

**Table 2:** Pre- and post-intervention comparison of the nutritional knowledge level and perceived severity between the groups*

	Before Intervention	After Intervention	T	P value
Knowledge				
Intervention	9.00±2.15	12.20±2.47	-7.90	0.001
Control	9.28±2.80	9.80±2.10	-1.30	0.210
T	- 0.45	4.28		
P value	0.652	0.001		
				
Perceived severity				
Intervention	18.75±4.40	21.80±4.30	- 2.80	0.007
Control	18.03±5.00	17.90±4.60	0.11	0.913
T	- 0. 61	3.54		
P value	0.541	0.001		

* Data are presented as means±SD or numbers (%)

**Table 3:** Pre- and post-intervention comparison of the perceived benefits and barriers and dietary behavior between the groups*

	Before Intervention	After Intervention	T	P value
Perceived benefits and barriers				
Intervention	14.60±3.40	17.80±4.70	- 3.12	0.003
Control	14.20±3.11	14.50±3.20	0.32	0.752
T	- 0.46	4.60		
P value	0.650	0.001		
				
Dietary behavior				
Intervention	27.00±3.10	27.30±3.22	- 0.88	0.394
Control	26.20±2.90	25.20±3.00	- 0.72	0.481
T	- 2.27	2.66		
P value	0.080	0.001		

* Data are presented as means±SD or numbers (%)

## Discussion

The results of the present study confirmed the remarkable role of an effective nutritional education program in the improvement of nutritional knowledge and also perceived severity and benefits among the patients under investigation. The results indicated a significant difference in nutritional knowledge among the CABG patients in the control and intervention groups after the intervention. On the other hand, a statistically significant difference was found in nutritional knowledge among the patients in the intervention group before and after the intervention (p value = 0.001). Our results corroborate the findings of a study by Zigheimat et al.,^9^ who reported that nutritional knowledge among the patients in the intervention group improved significantly after intervention. Sharifi Rad et al.^10^ found that education had a significant role in improving the nutritional knowledge of the patients in the intervention group. In a study by Adili et al.^11^ in Tehran, although more than half of the participants mentioned a sedentary lifestyle as an effective factor in CVD, only a quarter of them had a regular workout program. Also, although more than two-thirds of the participants were aware of the role of diet in CVD, less than half of them included sufficient amounts of fruit and vegetables in their diets. A comparison of perceived severity between the control and intervention groups showed that although before the intervention the difference was not statistically significant, a significant difference was seen between them after the intervention. Moreover, there was no significant difference in the perceived severity of the control group before and after the intervention; however, this difference was significant in the intervention group. The results of the present study are in line with the findings of a study by Zinat Motlagh et al,^12^ conducted based on the HBM. These findings showed the positive effects of education on improving perceived severity based on the HBM.

In our study, the mean scores of perceived benefits and barriers were not significantly different between the control and intervention groups before the intervention; however, after the intervention, the figures were significantly different between the 2 groups. Moreover, the mean scores of perceived benefits and barriers in the intervention group were significantly different before and after the intervention. Our results showed that education based on the HBM was effective in improving perceived benefits and barriers, which corresponds with the findings of studies by Shoja Fard et al.^13^ and Zinat Motlagh et al.^12^ The study by Zigheimat et al.^9^ also revealed a significant improvement in perceived benefits and barriers after intervention. Finally, although after the intervention the mean score of dietary behavior was significantly higher in the intervention group than in the control group (p value = 0.001), there was no significant difference before and after the intervention in the control (p value = 0.210) and intervention (p value = 0.394) groups. In a study by Shojaei Zadeh et al.^14^ on the application of the HBM in the prevention of osteoporosis, the consumption of calcium decreased in both control and intervention groups. Also in the study by Adili et al.,^11^ although the participants enjoyed a high level of knowledge regarding the risk factors of CVD, there was a deep gap between their knowledge and their behavior. Additionally, Mosayebi et al.^15^ confirmed the effect of education based on the HBM on preventive behaviors among primary school students in preventing parasitic infections.

In the current study, the absence of a significant difference in dietary behavior before and after the intervention in the intervention group showed that creating behavioral change is a difficult, time-consuming, and persistent process that requires a coherent long-term program. Lisspers et al.^16^ studied CABG patients with a history of acute myocardial infarction with a focus on health-related education and creating desirable changes in the patients’ lifestyle and found that after 1 year, the death and illness rates dropped substantially and the patients’ blood fat, sporting capabilities, body mass index, psychological variables, stress, diet, and smoking habits showed significant improvements. Also in the studies by Zigheimat et al.^9^ and Shoja Fard et al.,^13^ the dietary behavior in the intervention group showed a significant improvement. Considering the effect of knowledge on behavior as a determining factor, we can conclude that creating permanent change in individuals’ dietary behavior through health-related programs requires a great deal of background and involves people who have a role in the patients’ diet.

## Conclusion

The present study showed that although nutrition education based on the HBM had a significantly positive effect on nutritional knowledge, dietary behavior, perceived severity, and perceived benefits and barriers in the intervention group compared to the control group, it failed to create a significant change in the dietary behavior of the intervention group compared to that before the study. It shows that creating along-lasting change in individuals’ behavior requires consistent monitoring and inclusive programs through long-term interventions and involves the people who play a role in the patients’ diet and lifestyle. Moreover, significant differences before and after the intervention in nutritional knowledge, perceived severity, and perceived benefits and barriers between the 2 study groups showed that educational interventions had enhanced these factors in the patients. Perhaps the lack of attention to nutritional skills in educational programs based on the HBM was the reasons behind the absence of change in dietary behavior. Therefore, more attention should be paid to health-related and nutritional education of heart patients based on proper models. Further studies are required in this field because studies on CABG patients are mostly incomprehensive and far from all-inclusive.

## Notes:

***This paper should be cited as:*** Shojaei S, Farhadloo R, Aein A, Vahedian M. Effects of the Health Belief Model (HBM)-Based Educational Program on the Nutritional Knowledge and Behaviors of CABG Patients. J Teh Univ Heart Ctr 2016;11(4):181-186.
